# Low-cost potentiometric paper-based analytical device based on newly synthesized macrocyclic pyrido-pentapeptide derivatives as novel ionophores for point-of-care copper(ii) determination

**DOI:** 10.1039/d1ra04712d

**Published:** 2021-08-18

**Authors:** Ayman H. Kamel, Abd El-Galil E. Amr, Abdulrahman A. Almehizia, Elsayed A. Elsayed, Gaber O. Moustafa

**Affiliations:** Chemistry Department, College of Science Sokheer 32038 Kingdom of Bahrain ahkamel76@sci.asu.edu.eg aamr@ksu.edu.sa; Department of Chemistry, Faculty of Science, Ain Shams University Cairo 11566 Egypt; Pharmaceutical Chemistry Department, College of Pharmacy, King Saud University Riyadh 11451 Saudi Arabia mehizia@ksu.edu.sa; Applied Organic Chemistry Department, National Research Center Giza 12622 Egypt; Bioproducts Research Department, Zoology Department, Faculty of Science, King Saud University Riyadh 11451 Saudi Arabia eaelsayed@ksu.edu.sa; Chemistry of Natural and Microbial Products Department, National Research Centre Dokki 12622 Cairo Egypt; Department of Peptide Chemistry, National Research Centre Cairo Egypt gosman79@gmail.com

## Abstract

A simple, cost-effective, portable and disposable paper-based analytical device is designed and fabricated for copper(ii) determination. All solid-state ion-selective electrodes (ISEs) for copper and a Ag/AgCl reference electrode were constructed and optimized on the paper substrate. The copper electrodes were built using carbon nano-tube ink as a conductive substrate and an ion-to electron transducer. A suitable polymeric membrane is drop-cast on the surface of the conductive carbon ink window. The copper-sensing membrane is based on newly synthesized macrocyclic pyrido-pentapeptide derivatives as novel ionophores for copper detection. Under the optimized conditions, the presented all-solid-state paper-based Cu^2+^-ISEs showed a Nernstian response toward Cu^2+^ ions in 30 mM MES buffer, pH 7.0 over the linear range of 5.0 × 10^−7^–1.0 × 10^−3^ M with a limit of detection of 8.0 × 10^−8^ M. The copper-based sensors exhibited rapid detection of Cu^2+^ ions with a short response time (<10 s). The selectivity pattern of these new ionophores towards Cu^2+^ ions over many common mono-, di- and trivalent cations was evaluated using the modified separate solution method (MSSM). The presented paper-based analytical device exhibited good intra-day and inter day precision. The presented tool was successfully applied for trace Cu^2+^ detection in real samples of serum and whole blood collected from different children with autism spectrum disorder. The data obtained by the proposed potentiometric method were compared with those obtained by the inductively-coupled plasma (ICP) as a reference method. The presented copper paper-based analytical-device can be considered as an attractive tool for point-of-care copper determination because of its affordability, vast availability, and self-pumping ability, particularly when combined with potentiometric detection.

## Abstract 2

A simple, cost-effective, portable and disposable paper-based analytical device is designed and fabricated for copper(ii) determination.

## Graphical abstract

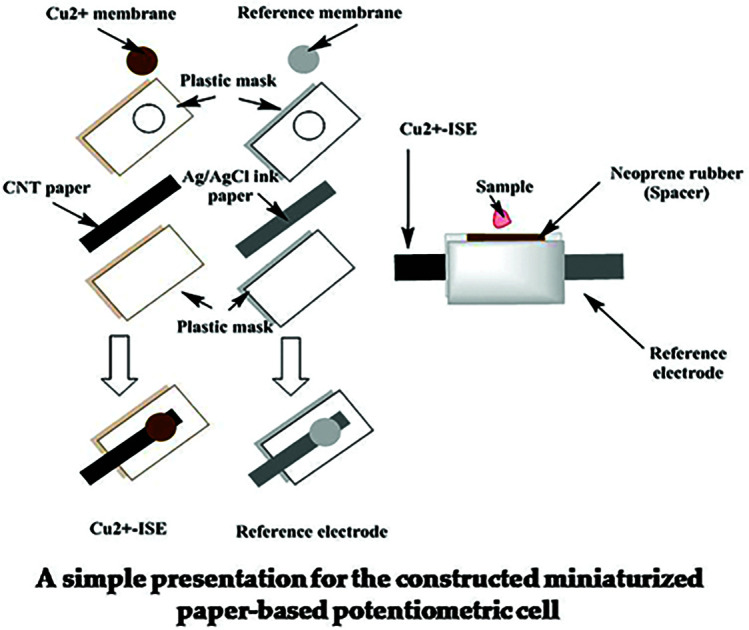

## Introduction

Paper-based analytical devices (PADs) have attracted widespread interest in the different fields of analytical chemistry since 2007 due to their multiple advantages over the traditional methods of analysis. Some of these advantages are cost-effectiveness during production, ease of portability, simplicity of operation, miniaturization, compatibility with biomolecules, low consumption of chemical reagents and high speed of detection at the point of care.^1–4^ Some PADs move fluids through capillaries without the need for external pumps and are therefore considered as alternative platforms for this purpose.^5^ Fabrication of ePADs often involves patterning paper to create fluidic channel(s), incorporation of electrodes and other detection components (*e.g.* catalysts and recognition elements), and assembly of multilayer devices for specific applications (*e.g.* vertical fluidic flow, fast fluidic flow, and multistep assays). Various fabrication methods to perform these tasks have been reported in the literature with wax patterning methods and the use of carbon-based electrodes among the most popular because of their low cost. Multiplexed analysis can be carried out through adding channels. The fabrication of such devices includes wax-printing, screen-printing, ink-printing, photolithography and oxidation by plasma.^6^ PADs have been widely used in various fields including clinical diagnosis, environmental monitoring, and food safety assurance using various electrochemical, color, fluorescence, immunological and molecular analytical methods.^5–14^

PADs were used to detect heavy metals, which is of great concern due to its toxicity to both humans and animals.^15^ Copper is one of the most predominant and widely used heavy metals, so its amount in environmental and industrial issues must be under control.^16^ It is also considered one of the micro-nutrients necessary for the organism because it is involved in the formation of a number of essential proteins. Copper deficiency in the organism leads to various diseases, such as bone deformity in children, osteoporosis in adults, and cardiovascular diseases.^17^ Despite this, copper is toxic in a high-level concentration and pose a risk. The harmful effect of copper can be seen in several diseases including abdominal pain, nausea, Alzheimer's disease, and Wilson's disease. Furthermore, an increased amount of copper in the body harms the kidneys and liver, and may also contribute to cancer formation.^18^ Taking the above factors into consideration, controlling the copper content is indispensable. So, the need for a credible, simple, cost-effective, fast and remote method for the determination of copper is of great particular interest in the point-of-care area.

There are several methods of copper determination reported in the literature. They include stripping voltammetery,^19–21^ fluorimetry,^22^ inductively coupled plasma/mass spectrometry (ICP/MS),^23^ atomic flame absorption spectrometry (AAS/flame),^24–26^ thermoelectric atomic absorption spectrometry (AAS/flameless),^27^ and chromatography.^28^ These reported approaches possess some merits such as reasonable selectivity and low-detection limits, but they are sophisticated, time-consuming, require highly expensive instruments, and are not suitable for point-of-care analysis. Therefore, these techniques are not suitable for on-line detection and daily-control of copper content in different fields. These limitations can be overcome by using potentiometric sensors.^29–32^

All-solid-state potentiometric sensors, wherein a solid-contact layer is inserted between the electrode substrate and an ion-sensing membrane (ISM), act an important role in the detection. These types of electrodes possess good merits such as high potential stability, ease of construction, cost-effective and ability of miniaturization. The application of these electrode design offers fast analysis, short-response time, good selectivity and low-cost analysis.^33–35^ All of these merits make potentiometric approaches the most favorable approach for Cu^2+^ assessment. Although there are many solid electrodes for copper measurement in the literature,^36–45^ they have been applied in environmental analyses. There are no reported sensors for copper determination based on the paper as a solid support for point-of-care purposes.

Herein, a disposable paper-platforms based potentiometric micro-cell was developed for rapid, reliable and accurate assessment of copper ions in whole blood. Novel paper-based copper sensors based on newly synthesized macrocyclic pyrido-pentapeptide derivatives as novel ionophores for copper detection were built and characterized. Then, a novel paper-based solid-state reference electrode is integrated with the constructed copper sensor to build up the potentiometric-cell with an approximately total volume of ∼50 μL. Factors affecting the analytical performance of the presented potentiometric cell were characterized and discussed. The device was successfully applied for accurate determination of copper in whole blood samples collected from autistic children. The data were compared with those obtained by ICP/OES method, and showed no significant difference at 95% confidence interval. The presented potentiometric device opens new avenues for managing copper and implementing paper-based analytical platforms.

## Experimental

### Chemicals and reagents

Potassium tetrakis(4-chlorophenyl)borate (KTClPB), tridodecylmethylammonium chloride (TDMAC), 2-nitrophenyl octyl ether (NPOE, purity >99%), fluorinated alkyl silane (CF_3_(CF_2_)_7_CH_2_CH_2_SiCl_3_, C^F^_10_) high molecular weight polyvinyl chloride (PVC), tetrahydrofuran (THF), poly vinyl butyral (PVB), CH_3_OH (purity 99.8%) and 2-(*N*-morpholino)ethanesulfonic acid (MES) were all purchased from Sigma-Aldrich (St Louis, Missouri, MO, USA). All salts of Cu^2+^, Fe^3+^, NH_4_^+^, Pb^2+^, Ag^+^, Hg^2+^, Sn^2+^, Al^3+^, Zn^2+^ and Cd^2+^ were of analytical grade, and in the form of either nitrate, chloride or sulfate salts and were purchased from Sigma-Aldrich. Ag/AgCl ink (E2414) was purchased from Ercon (Wareham, MA). Conductive-carbon ink was purchased from Bohui New Materials Tech. Co. Ltd (Jiangsu, China). Milli-Q PLUS deionized water (18.2 MΩ cm^−1^) (Millipore Corporation, Bedford, MA, USA) was used for all solutions preparation. Artificial serum samples were prepared after dissolving 111 mM of NaCl, 29 mM of NaHCO_3_, 2.2 mM of K_2_HPO_4_, 0.8 mM of MgCl_2_, 2.5 mM of urea and 4.7 mM of glucose.^46^

All serum and blood samples were collected from different patients have autism disorders and were provided by a local Egyptian hospital.

### Instrumentation

All potentiometric measurements were carried at room temperature (22 °C) using mV/pH meter (PXSJ-216, INESA Scientific Instrument Co., Ltd, Shanghai, China). A double-junction Ag/AgCl/KCl 3 M/1 M LiAcO reference electrode (Metrohm AG 6.0726.100) was used for optimizing and comparing the developed paper-based reference electrode. ICP-OES (CAP 6000 series ICP-OES, Thermo Scientific, USA) was used for the determination of Cu(ii) in real samples to obtain reference values compared to those obtained by the presented potentiometric method.

### Ionophores synthesis

**Fig. 1:**
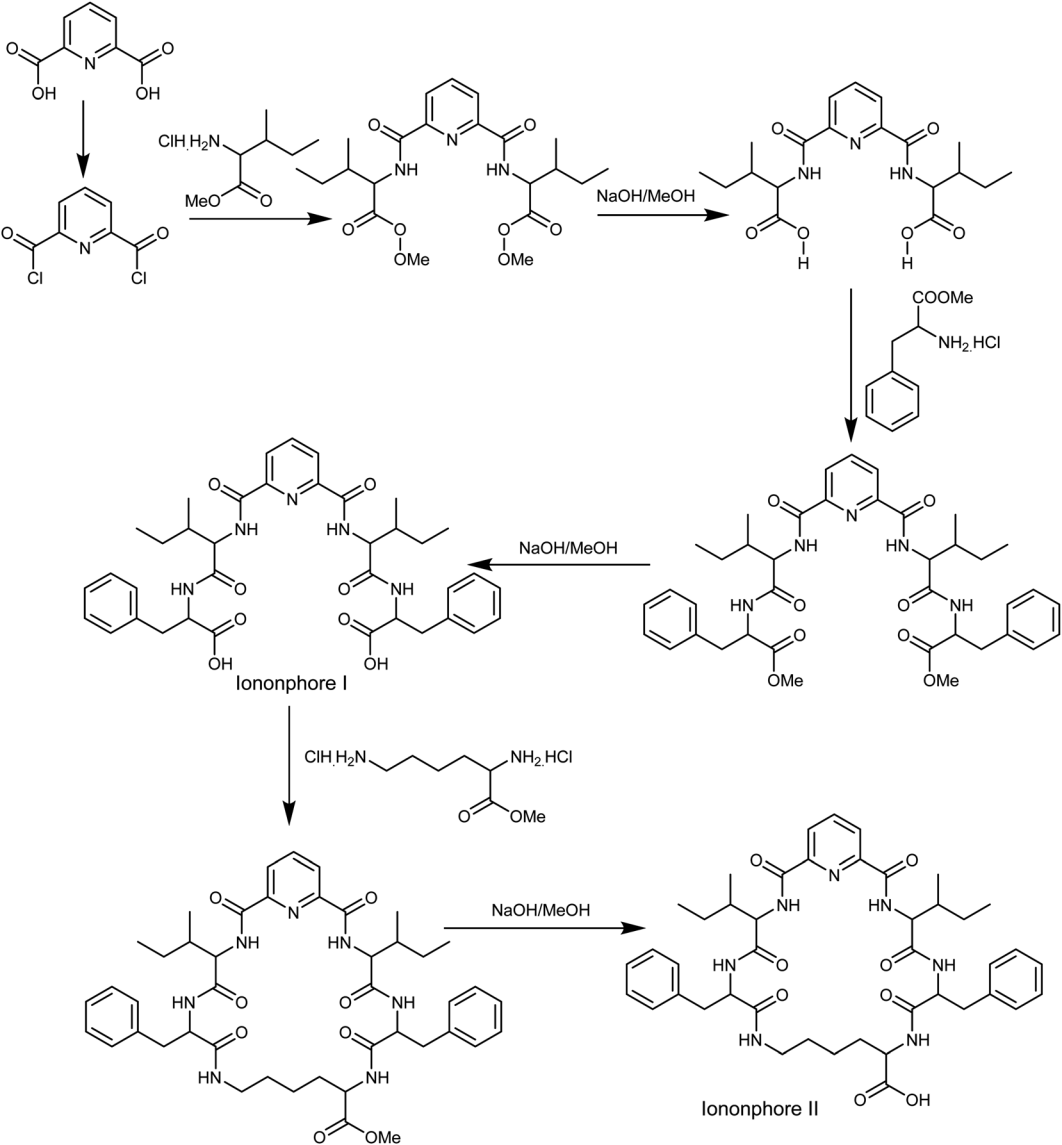
Synthetic pathway of copper ionophores.

The synthesis of macrocyclic pyrido-pentapeptide derivatives shown here as copper ionophores (Fig. 1) were prepared and elucidated according to literature procedures.^47^ In brief, the methyl ester of l-amino acid was initially coupled with the acid chloride of dipicolinic acid to produce *N*,*N*-bis-[1-carboxy-2-(benzyl)]-2,6-(diamino-carbonyl) pyridine. The product is then treated with l-amino acid methyl ester hydrochloride in the presence of ethyl chloroformate in dichloromethane afforded the corresponding tetrapeptide pyridine methyl ester derivatives. Hydrolysis with methanolic sodium hydroxide affords ionophore I. Cyclization was carried out using l-lysine methyl ester to afford the corresponding cyclic pentapeptide ester. This compound is then hydrolyzed with methanolic sodium hydroxide to give the corresponding cyclic pentapeptide acid (Fig. 1).

### Cell design and fabrication

A qualitative filter-paper was used as a supporting substrate. To make the paper hydrophobic, the paper was inserted in a Petri-dish containing 20 mL C^F^_10_. In a drying chamber, the solvent was evaporated at 80 °C for 30 min until a uniform layer of CF 10 is formed above the paper substrate.^48^ The carbon-nanotube ink (CNT) is then painted above the hydrophobic paper substrate and left to dry in the oven for 20 min. The measured resistance of the paper was around 300 Ω sq^−1^ and becomes conductive. The paper is then covered with a plastic mask (0.3 mm thick), leaving a window (2.0 mm) where the ion-sensing membrane is then drop-cast. The copper-sensing membrane was prepared by dissolving 2.0 mg of the ionophore, 1.0 mg of potassium tetrakis(4-chlorophenyl)borate (KTClPB), 28.5 mg of polyvinylchloride (PVC) and 68.5 wt% of 2-nitrophenyl octyl ether (NPOE) in 1.5 mL THF. To construct the solid-state reference electrode, Ag/AgCl ink was coated on the hydrophobic paper and left to dry then covered with a plastic mask leaving a window width 2 mm. The reference membrane was prepared by dissolving 28 mg of NaCl, 28.0 mg of AgNO_3_ and 44.0 mg of PVB in 1 mL of methanol.^49^ 20 μL of both copper-sensing membrane and reference membrane were drop-cast on their respective electrodes, 5 μL at a time.

**Fig. 2:**
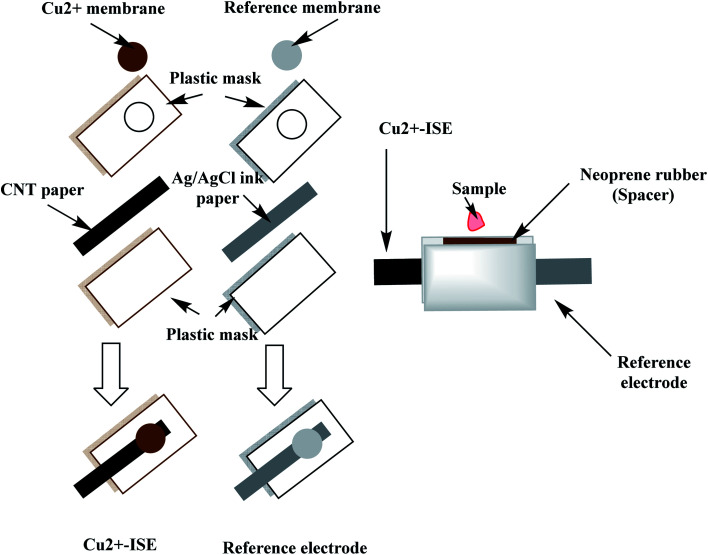
A simple presentation for the constructed miniaturized paper-based potentiometric cell.

The miniaturized cell was built up by sandwiching the two electrodes leaving a cavity of ∼50 μL volume using neoprene rubber of 3 mm thick. The constructed paper-based potentiometric device is then connected to the mV/pH meter through the conductive ends of both working and reference electrodes. A simple presentation for constructing the miniaturized cell is shown in Fig. 2.

For sensors based on glassy carbon (GC) substrates, a GC disk electrode (4 mm I.D.) were polished by 0.3 μm γ-Al_2_O_3_ and sonicated with ethanol and de-ionized water alternatively and then dried under N_2_ stream. A piece of PVC tube (1 cm length, 5 mm I.D. and 8 mm O.D.) was inserted at the distal end of the GC substrate. A 10 μL of CNTs ink was coated above the GC disk. After drying, the electrodes were washed with de-ionized water and then dried under a stream of N_2_ gas. A 100 μL volume of the membrane cocktail (*i.e.* the same composition as mentioned above) was drop-casted above the CNTs layer. Afterward, the membrane was left to dry until a uniform shape is obtained with good adhesion to the GC substrate.

## Results and discussion

### Characterization of copper paper-based analytical device

**Fig. 3:**
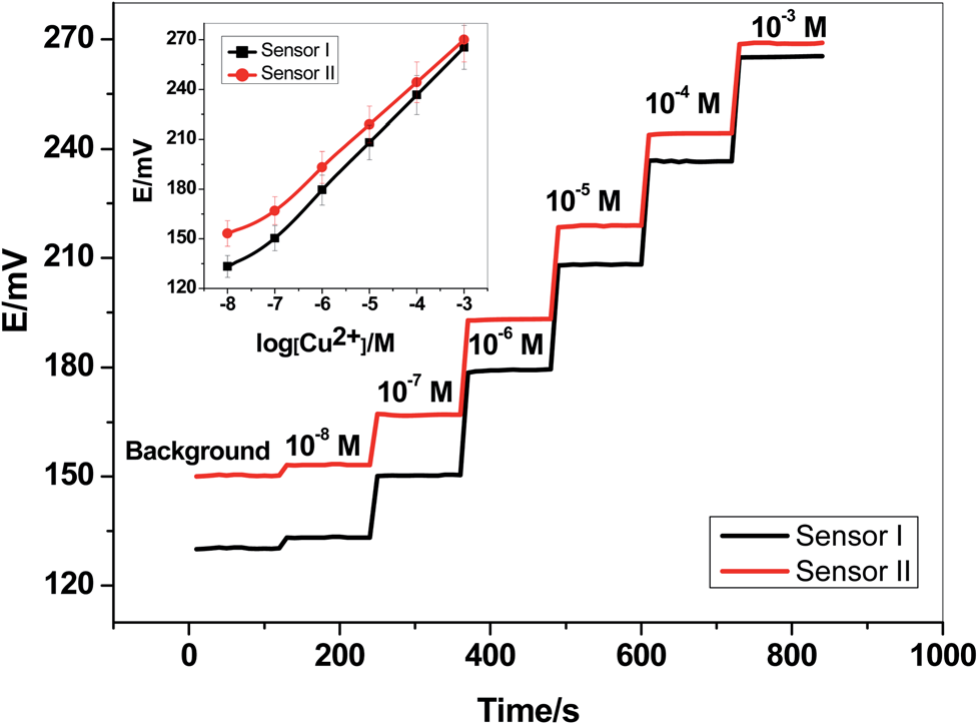
The potentiometric response and time-trace for Cu^2+^-paper based sensors.

Two newly synthesized macrocyclic pyrido-pentapeptide derivatives were synthesized and used as novel neutral-carriers for Cu^2+^ ions in plasticized PVC matrix. The potentiometric paper-based analytical device includes both the copper sensor and the reference Ag/AgCl electrode. Five paper-based devices for each ionophore were constructed and their analytical performances were evaluated according to IUPAC- recommendations.^50^ The presented potentiometric paper-based devices have a potentiometric response shown in Fig. 3. The obtained linearity range for sensors based on ionophore I (sensor I) and ionophore II (sensor II) was 5.0 × 10^−7^–1.0 × 10^−3^ and 4.0 × 10^−7^–1.0 × 10^−3^ M with slopes of 28.6 ± 0.5 and 25.6 ± 0.8 mV per decade and detection limits of 8.0 × 10^−8^ and 6.5 × 10^−8^ M, respectively.

For electrode optimization and comparison purposes, Cu^2+^-ISEs based on glassy-carbon (GC-ISE) support were also constructed and their results were compared with the paper-based analytical devices. The GC/Cu^2+^-ISEs based on ionophore I (sensor III) and ionophore II (sensor IV) showed a Nernstian response with slopes of 29.1 ± 0.5 and 25.6 ± 0.2 mV per decade (30 mM MES buffer, pH 7.0) over the linear range 1.0 × 10^−7^–1.0 × 10^−3^ with a limit of detection of 3.4 × 10^−8^ and 3.4 × 10^−8^ M, respectively. The obtained results are very similar to those obtained by the presented Cu^2+^-paper based analytical devices.

**Fig. 4:**
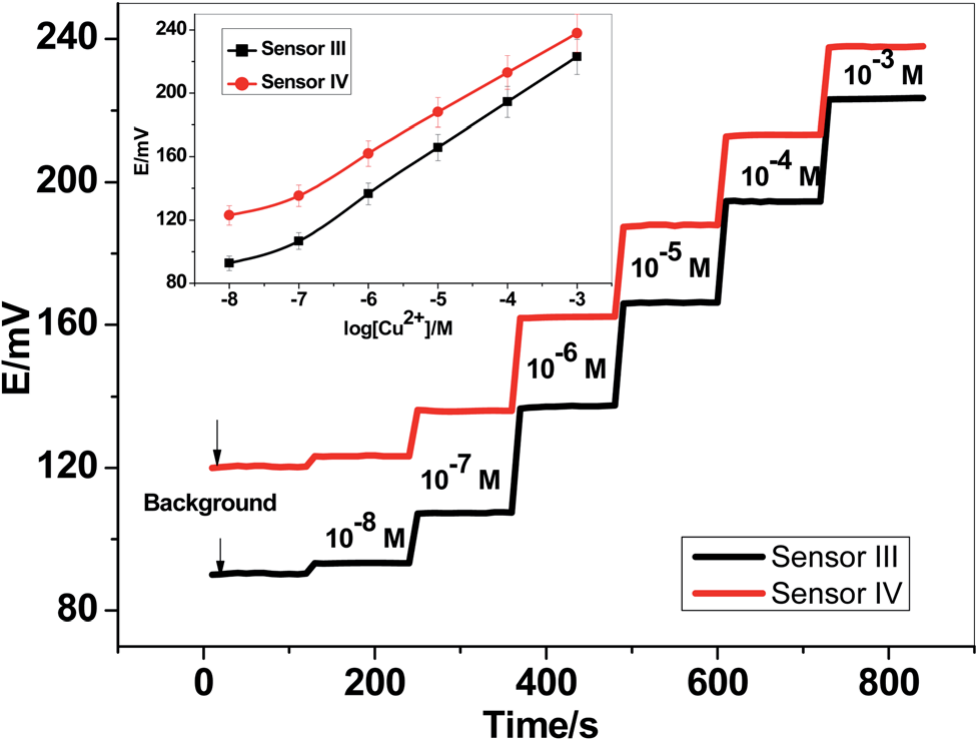
The potentiometric response and time-trace for GC/Cu^2+^-based sensors.

The calibration plots for GC/Cu^2+^-ISEs based on ionophores I and II are shown in Fig. 4. This shows that, there are no significant differences between the presented paper-based analytical devices and the conventional solid-state GC/Cu^2+^-ISEs in terms of slope-sensitivity and linearity-range. The time-trace response of both paper-based analytical devices and solid-state GC/Cu^2+^-ISEs based on ionophores I and II are shown in Fig. 3 and 4, respectively. The sensors attained a steady-state potential response in less than 10 s, which is good and reasonable for the use of these devices in de-centralized analysis. A long-term potential stability test showed a potential drift of about 0.3 and 0.25 mV h^−1^ (16 h) for sensors I and II, respectively, and which is also satisfactory, considering that these devices are intended for a single-short reading. The performance analytical characteristics are summarized in Table 1.

**Table 1:** Performance potentiometric characteristics of Cu^2+^-sensors

Parameter	Sensor I	Sensor II	Sensor III	Sensor IV
**Slope (mV per decade)**	28.6 ± 0.5	25.6 ± 0.8	29.1 ± 0.6	25.6 ± 0.2
**Correlation coefficient (*r*** ^**2**^ **)**	0.999	0.999	0.999	0.999
**Linear range (M)**	5.0 × 10^−7^–1.0 × 10^−3^	4.0 × 10^−7^–1.0 × 10^−3^	1.0 × 10^−7^–1.0 × 10^−3^	1.0 × 10^−7^–1.0 × 10^−3^
**Detection limit (M)**	8.0 × 10^−8^	6.5 × 10^−8^	3.4 × 10^−8^	3.3 × 10^−8^
**Working acidity range (pH)**	4.0–7.5	4.0–7.5	4.0–7.5	4.0–7.5
**Response time (s)**	<5	<5	<5	<5
**Accuracy (%)**	98.2	98.7	98.8	97.9
**Trueness (%)**	99.2	99.3	98.7	98.6
**Bias (%)**	0.6	0.4	0.9	1.1
**Intra-day precision (%)**	0.8	1.3	1.1	0.9
**Inter-days precision (%)**	1.1	0.8	0.9	1.2

Intra-day and inter-day precision were examined for the presented paper-based analytical devices. 1.0 μg mL^−1^, internal quality control sample of copper was measured (*n* = 6). The relative standard deviations were found to be 0.8 and 1.3 for sensors I and II, respectively. Method accuracy was also evaluated by spiking a known Cu^2+^ amount (0.5 μg mL^−1^) and found to be 98.2 ± 0.7–98.7 ± 0.6% for sensors I and II, respectively.

The effect of pH on the potential response was tested. The presented sensors showed good stability over the pH range of 4 to 7.5, the electrode-potential does not change by more than ±0.8 mV. At pH values >8 the potential begins to decline at concentrations >10^−4^ M due to the formation of precipitation of Cu(OH)_2_ and/or the formation of copper-hydroxo complexes. At pH < 4, the electrode potential increases due to some interferences coming from H^+^ ions. All subsequent-potentiometric measurements of Cu^2+^ ions in blood or serum were made as the sample received or in 30 mM MES buffer background of pH 7.0.

### Interfering ions study

**Fig. 5:**
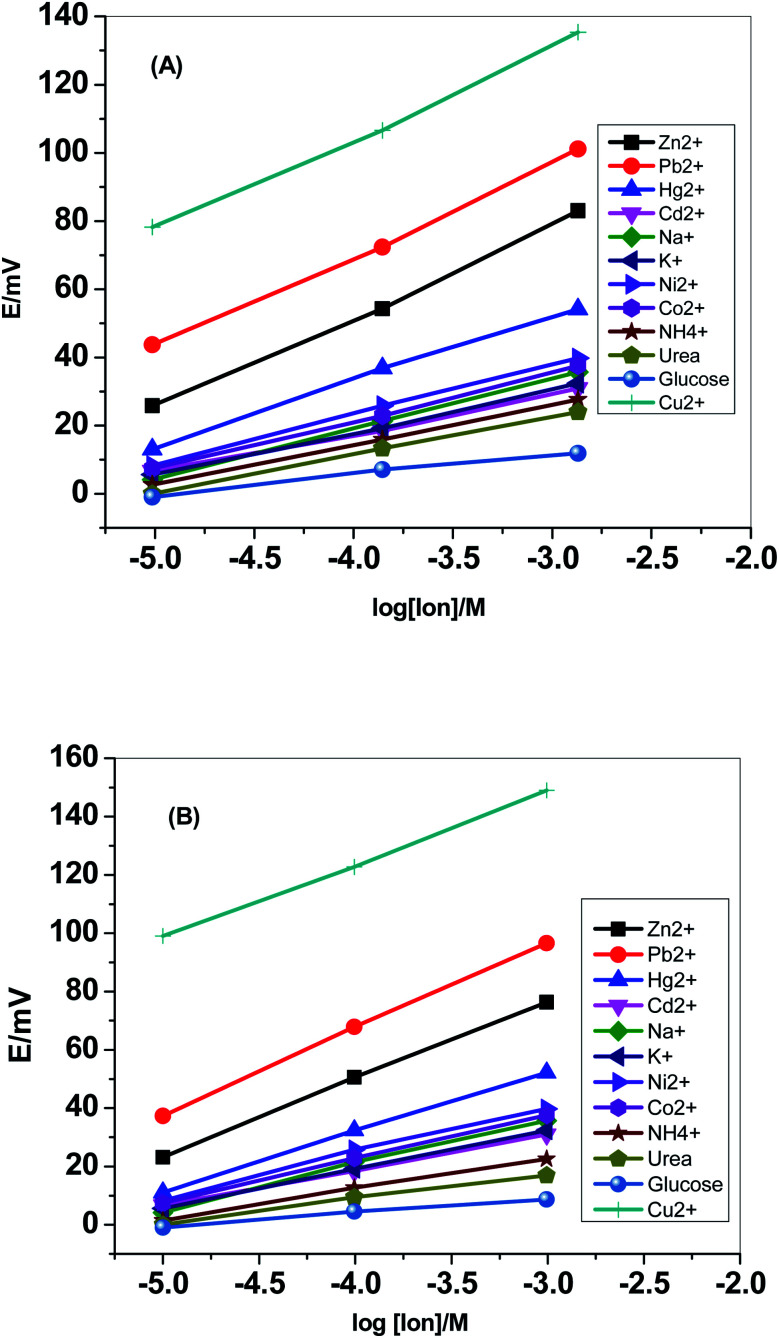
Selectivity pattern for the paper-based electrochemical device based on (A) sensor I and (B) sensor II.

Selectivity test was carried out using the modified separate solution method (MSSM).^51,52^ Successive calibration-curves with increasing the concentrations of the interfering ions and the last calibration was carried out with Cu^2+^ ions. The potentiometric selectivity values were calculated by inserting the extrapolated potentials of each curve at 1 M concentration into the SSM equation. The selectivity pattern for the paper-based electrochemical device based on both sensor I and II is shown in Fig. 5.

**Fig. 6:**
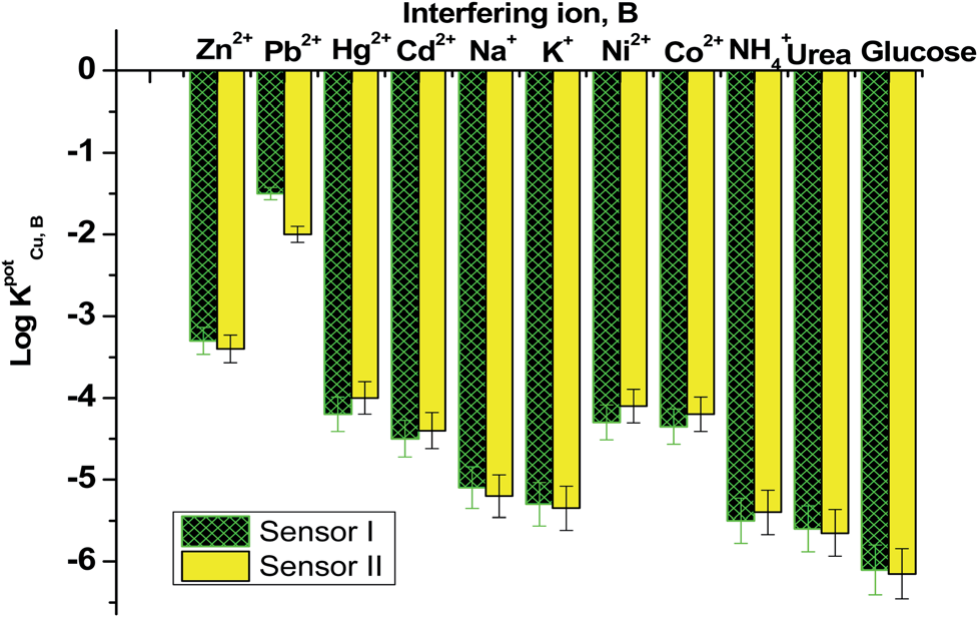
Selectivity coefficient pattern for copper-paper selective electrodes based on ionophores I and II.

Fig. 6 displayed the selectivity coefficient values (log *K*^Pot^_Cu^2+^_,_B_) for the most abundant ions and organic compounds that can be found in blood. It was noticed that ionophore I displayed better selectivity towards Cu^2+^ ions over Hg^2+^, Cd^2+^, Ni^2+^, Co^2+^ and NH_4_^+^ ions than ionophore II. Both ionophores revealed nearly the same selectivity behavior over K^+^, urea and glucose. Ionophore II exhibited better selectivity towards copper ions over Zn^2+^, Pb^2+^ and Na^+^ ions than ionophore I. From the data presented in Fig. 6, it could be expected that the presented ionophores have high selectivity towards copper ions and its applicability in determining copper content in whole blood without major interferences could be successfully.

### Copper assessment in serum and blood samples

The paper-based potentiometric devices were applied for copper ions assessment at first, in artificial-serum samples and then in real human-serum samples. The normal level of copper was reported to be 70–140 μg dL^−1^.^53^ All samples were analyzed in triplicate, and the mean of three measurements was presented. Different Cu^2+^ concentrations were added to the samples covering the range of 0.5–10.0 μg mL^−1^. As shown in Table 2, the recovery range was found to be 92.0–105.0% and 94.0–105.0% for sensors I and II, respectively.

**Table 2:** Copper determination in spiked artificial serum samples using copper paper-based potentiometric devices

Sample no.	Added, μg mL^−1^	Sensor I		Sensor II	
		Found^a^, μg mL^−1^	Recovery, %	Found^a^, μg mL^−1^	Recovery, %
1	0.5	0.46 ± 0.04	92.0	0.51 ± 0.03	102.0
2	2.0	2.1 ± 0.3	105.0	2.1 ± 0.4	105.0
3	5.0	4.7 ± 0.5	94.0	4.8 ± 0.2	96.0
4	10.0	9.7 ± 0.2	97.0	9.4 ± 0.5	94.0

a Average of 5 measurements.

To check the applicability of the presented paper-based potentiometric device for copper determination, six serum samples were collected from different children have autism disorders and then analyzed. Before sample analysis, three standard solutions of copper (*e.g.*, 10.0, 50.0 and 100.0 μg mL^−1^) were inserted into the potentiometric-cell to build-up the calibration plot. After measuring the standard calibrants, the cell is then washed and the sample is analyzed. The same samples were analyzed with ICP-OES as a reference method-that is normally used in the routine analysis of copper in blood and serum. The results of measuring both serum and blood samples were shown in Tables 3 and 4, respectively. The data confirmed that the analysis was of acceptable accuracy when compared with those obtained by the standard ICP-OES method.

**Table 3:** Copper determination in real serum samples collected from autistic children

Sample no.	Copper content^a^, μg mL^−1^		
	Potentiometry		ICP-OES
	Sensor I	Sensor II	
**Male (age 5–8 years)**			
1	19.4 ± 12	17.2 ± 9.0	16.2 ± 0.8
2	57.3 ± 5.0	52.2 ± 3.0	55.6 ± 0.2
3	44.5 ± 0.7	39.2 ± 6.0	42.2 ± 0.1

**Female (age 4–8 years)**			
4	55.2 ± 0.9	51.9 ± 4.0	54.4 ± 0.3
5	63.2 ± 2.0	61.5 ± 6.0	67.2 ± 0.5
6	48.1 ± 4.0	51.2 ± 3.0	45.3 ± 0.1

a Average of 3 measurements.

**Table 4:** Copper determination in blood samples collected from autistic children

Sample no.	Copper content^a^, μg mL^−1^		
	Potentiometry		ICP-OES
	Sensor I	Sensor II	
**Male (age 5–8 years)**			
1	39.2 ± 0.9	36.1 ± 4.0	35.1 ± 0.2
2	37.3 ± 0.8	32.5 ± 2.1	35.6 ± 0.1
3	40.2 ± 1.7	37.2 ± 6.3	42.2 ± 0.3

**Female (age 4–8 years)**			
4	35.3 ± 1.9	31.2 ± 4.4	34.2 ± 0.1
5	33.4 ± 3.3	31.6 ± 1.4	37.5 ± 0.3
6	43.3 ± 3.6	41.4 ± 2.1	40.4 ± 0.2

a Average of 3 measurements.

## Conclusions

Herein, we presented a successful development of a paper-based potentiometric cell for the copper determination in biological fluids. The copper-based sensors are based on a newly synthesized macrocyclic pyrido-pentapeptide derivatives as novel ionophores for copper detection. These new ionophores exhibited high affinity towards copper detection within the linearity range 5.0 × 10^−7^–1.0 × 10^−3^ and 4.0 × 10^−7^–1.0 × 10^−3^ M with a sensitivity 28.6 ± 0.5 and 25.6 ± 0.8 mV per decade and a detection limit of 8.0 × 10^−8^ and 6.5 × 10^−8^ M for ionophores I and II, respectively. Merits and limitations of previously reported all-solid-state potentiometric copper electrodes in comparison with the presented sensors are presented in Table 5. The presented device is extremely simple to design, cost-effective, reliable, and have fast-response. The cell was successfully applied for the determination of copper content in blood and serum samples collected from autistic children. The results obtained were compared with those obtained by ICP-OES. The presented device offered different attractive features for point-of care analysis. Some of these merits are using low-volume sample (∼50 μL), working on the whole blood (without pre-treatment) and it is relatively fast. Therefore, this work can be considered as a good addition to the growing field of paper-based analytical platforms in point-of-care testing.

**Table 5:** Comparison of previously reported all-solid-state copper potentiometric electrodes with the presented paper-based electrochemical analytical device

Ionophore	Type of electrode	Solid contact transducer	Slope, mV per decade	Detection limit, M	Linear range, M	Ref.
(4-Phenyl-11-decanoyl-1,7-dithia-11-aza-cyclotetradecane-4-sulfide)	Pt micro-electrode	PPy [3,3′-Co (1,2-C_2_ B_9_ H_11_)_2_]	29.5 ± 1	5.6 × 10^−7^	1 × 10^−6^–1 × 10^−2^	36
Copper ionophore (IV)	GC	Graphene/7,7,8,8-tetracyano-quinodimethane	30.5 ± 0.05	8 × 10^−10^	1 × 10^−9^–1 × 10^−2^	37
Copper ionophore (II)	Graphite powder/resin	Three-dimensional graphene sponge	28.9 ± 0.7	2.5 × 10^−9^	1 × 10^−8^–7.9 × 10^−4^	38
Phytic acid/Ag composite	Gold substrate	Ag nanoparticles	31.1 ± 1.8	2.7 × 10^−6^	1 × 10^−5^–1 × 10^−3^	39
2-Mercapto-benzoxazole	Screen-printed	PEDOT/PSS	28	3 × 10^−7^	1 × 10^−6^–1 × 10^−2^	40
5-Sulfosalicylic acid	Pencil graphite	Polypyrrol	29.6 ± 0.3	5.4 × 10^−6^	1 × 10^−5^–1 × 10^−1^	41
Copper ionophore (ETH 1062)	Gold wire	PEDOT/PSS	28.1 ± 1.8	4 × 10^−8^	2.5 × 10^−7^–2.5 × 10^−4^	42
2-(1′-(4′-(1′′-Hydroxy-2′′-naphthyl)methyleneamino)butyl iminomethyl)-1-naphthol	Graphite powder/resin	MWCNTs	29.7 ± 0.2	5.5 × 10^−9^	1 × 10^−8^–1 × 10^−3^	43
Diphenylisocyanate bis (acetylacetone)ethylenediimine	Graphite powder/resin	MWCNTs	29.4 ± 0.4	2.4 × 10^−9^	1 × 10^−8^–1 × 10^−3^	44
*N*-Hydroxy succinamide	Graphite powder/resin	—	37.5	4.4 × 10^−6^	1 × 10^−4^–1 × 10^−2^	45
Macrocyclic pyrido pentapeptide derivative 1 (ionophore I)	Paper based modified with CNTs	Carbon nanotubes ink (CNTs)	28.6 ± 0.5	8 × 10^−8^	5.7 × 10^−7^–1 × 10^−3^	This work
Macrocyclic pyrido pentapeptide derivative 2 (ionophore II)			25.6 ± 0.8	6.5 × 10^−8^	4 × 10^−7^–1 × 10^−3^	

## Conflicts of interest

There are no conflicts to declare.

## Supplementary Material
